# Into the Unknown: Microbial Communities in Caves, Their Role, and Potential Use

**DOI:** 10.3390/microorganisms10020222

**Published:** 2022-01-20

**Authors:** Katarzyna Kosznik-Kwaśnicka, Piotr Golec, Weronika Jaroszewicz, Daria Lubomska, Lidia Piechowicz

**Affiliations:** 1 Department of Medical Mirobiology, Faculty of Mecicine, Medical University of Gdańsk, Dębowa 25, 80-204 Gdansk, Poland; katarzyna.kwasnicka@gumed.edu.pl; 2 Laboratory of Phage Therapy, Institute of Biochemistry and Biophysics, Polish Academy of Sciences, Kładki 24, 80-822 Gdansk, Poland; 3 Department of Molecular Virology, Institute of Microbiology, Faculty of Biology, University of Warsaw, Miecznikowa 1, 02-096 Warsaw, Poland; piotr.golec@uw.edu.pl; 4 Department of Molecular Biology, Faculty of Biology, University of Gdansk, Wita Stwosza 59, 80-308 Gdansk, Poland; weronika.jaroszewicz@ug.edu.pl (W.J.); darialajn@gmail.com (D.L.)

**Keywords:** Actinobacteria, caves, ecosystems

## Abstract

Caves have been an item of amateur and professional exploration for many years. Research on the karst caves has revealed great diversity of bacteria, algae, and fungi living on stone walls and speleothems, in mud puddles or sediments. They have become the source of interest for various research groups including geologists, chemists, ecologists, or microbiologists. The adaptations of cave-dwelling organisms applied to their survival are complex and some of their properties show potential to be used in various areas of human life. Secondary metabolites produced by cave’s bacteria show strong antimicrobial, anti-inflammatory, or anticancer properties. Furthermore, bacteria that can induce mineral precipitation could be used in the construction industry and for neutralization of radioisotopes. In this review we focus on bacteria and algae present in cave ecosystems, their role in shaping such specific environment, and their biotechnological and medical potential.

## 1. Introduction

There are a large number of caves on Earth. They have been used by both animals and humans for a long time for a variety of reasons. Archeological studies have shown evidence of caves being used by man since the Paleolithic period. Mainly, but not exclusively for shelter and storage, but also as places for artistic expression, burial, or ceremonial sites [1,2]. Today, caves are mainly objects of tourist activity, with people visiting show caves to admire Paleolithic paintings or interesting rock formations [3,4]. Furthermore, some caves are explored by adventurers during caving [5]. However, despite the amount of amateur and professional caving going on all over the world, it is estimated that only a tenth of the global number of caves have been discovered and described [6]. Hardly accessible, the farthest parts of the caves sparked an interest not only in speleologists but also scientists. Stable conditions of deep dark cave zones, specific to given caves, have resulted in the formation of closed, self-dependent ecosystems [6,7]. The studies on those ecosystems have revealed an abundance of various life forms, some of them never seen before. The interest in these life forms, especially microorganisms, has resulted in the discovery of new bacterial species and compounds. Some of them show medical or biotechnological potential due to antimicrobial activity or induction of mineral precipitation [6,8]. However, difficulty in recovery and isolation of these microorganisms brings into question whether the search for new compounds in caves is a worthy endeavor [6,9]. Nevertheless, with more knowledge about cave organisms and popularization of metagenomics studies, we observe an increased interest in microorganisms from extreme environments—caves included. This review focuses on knowledge about the properties of cave microorganisms (bacteria and algae) obtained in recent years, the role they play in cave ecosystems, and their biotechnological and medical potential.

## 2. Cave as a Specific Habitat

**Figure 1:**
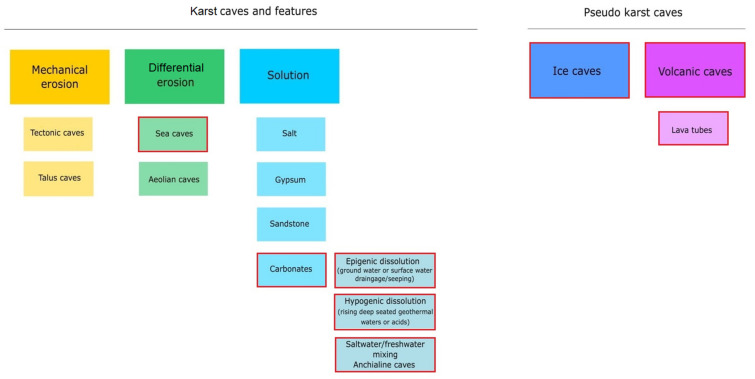
Karst and pseudo-karst caves formation processes and the types of caves that are formed as a result. Popular types of caves for microbiology studies are marked with red boxes (Cheeptham 2013, modified).

A cave is defined as a void in the ground, generally with an entrance large enough for a human to enter. In most cases they are formed in the process of speleogenesis, when the water with carbon dioxide dissolved within it produces carbonic acid, which permits the dissociation of the calcium carbonate in the rock (usually limestone or dolomite). Over time, the cracks enlarge and form caves and cave systems [10]. However, sometimes caves can be created in other ways, such as lava caves, which are formed as a result of solidification of a lava flow during the last stages of its activity, while littoral cave formation is a result of sea waves eroding the coastline [11] (Figure 1). Caves can be divided into four main zones based on the amount of light they receive [12]. The entrance zone—the area directly below the cave entry and next to the twilight zone—are the areas where light shines regularly, though the amount may vary depending on the season. These areas can be occupied by small, green vegetation [12,13]. The transition zone is the area receiving no direct light and is located between the twilight and the deep dark zone. The deep dark zone, the last one in the cave, receives no sunlight at all. It is also the area of the cave where the temperature, humidity, and CO_2_ pressure change little over the seasons and are stable regardless of the conditions outside the cave [7,13]. This results in each cave being unique in terms of biological, chemical, and physical characteristics, with little to no changes in these properties over time [6,7]. Furthermore, each cave zone can have its own ecosystem due to different life conditions [6,14]. The availability of sunlight, water, nutrients, the air flow, and the exposure to interactions with organisms living outside the caves are different in each cave zone and they influence the microflora that can develop and survive there [6,15].

**Figure 2:**
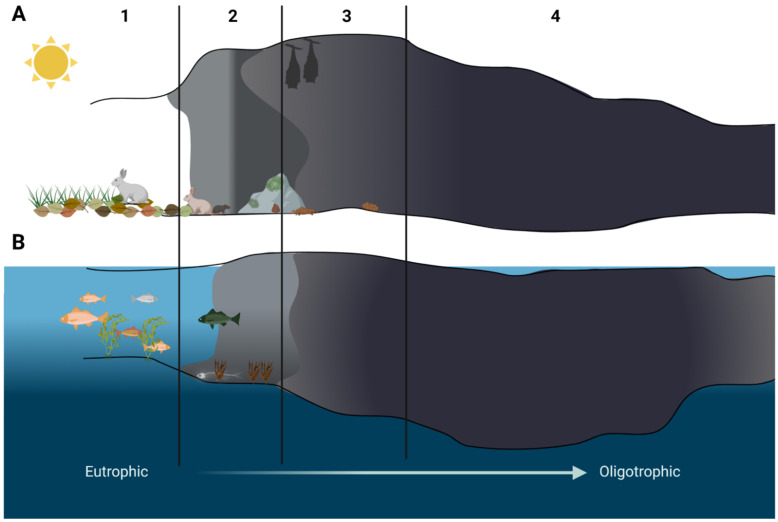
Schematic representation of cave zones in ground (**A**) and anchialine (**B**) caves. 1—entrance zone, 2—twilight zone, 3—transition zone, 4—dark zone. Source: Ghosh at al., 2017 (modified) and Calderón-Gutiérrez et al., 2018 (modified). Created with BioRender.com (accessed on 4 January 2022).

Anchialine caves and pools are landlocked and underground caves and cave systems filled with water and with a subterranean connection to the ocean. These extreme habitats are additionally influenced by tides and changes in sea levels, temperature, and composition [14,16]. However, different ecological zones have also been observed in those caves, but instead of light availability they are shaped by the distance from the open sea. The area close to the cave opening shows the highest species diversity, which diminishes as the distance to the sea increases [14]. Ecological zones of both ground and anchialine caves are presented in Figure 2. However, in this review we focus mainly on life conditions and ecological zones inside karst caves formed on land in carbonate rock (Figure 1) and the microorganisms inhabiting those.

Photosynthesizing lichens and algae were reported to live on the cave walls in the cave entrance zone [17,18]. This most external of the cave zones is also most likely to be influenced by visitors from outside: animals and humans [6,15,17]. Organic matter left behind such as feces or food scraps enrich the environment, increasing the amount of nutrients otherwise unavailable anywhere else inside the cave [19]. In the twilight zone, the amount of autotrophic organisms is lower. As the amount of light decreases we observe a shift towards heterotrophic lifestyle [6]. This zone can also be occasionally visited by animals from the outside, with these visits leaving its mark on the cave microbiome (Figure 2).

The influence of “outsiders” on the cave ecosystem is especially visible in so-called “show caves”, where large groups of tourists arrive daily [15,17,20]. Constant human presence contributes to changes in humidity and temperature. The litter left behind can have a negative impact not only on animals inside the caves, but on microorganisms as well. However, the most visible negative influence on the ecosystem is the influence of lamps used in caves to display Paleolithic art or interesting rock formations [15]. The lamps enable phototrophic organisms to form biofilms close to the light source, destroying the art in the process and disrupting the balance in the environment [15,21].

The dark, oligotrophic, and generally inhospitable environment of the most internal zone of the cave was for a long time perceived to be deprived of life forms. However, zoological and botanical studies showed over time a vast diversity of highly specialized lifeforms thriving inside various caves all over the world [22,23,24]. Furthermore, an interest in cave microbiome has been observed, as microorganisms isolated from these environments were revealed to have interesting properties in biotechnological, medical, and ecological sense [6,25,26].

## 3. Microorganisms in Cave Ecosystems

Despite unfavorable growth conditions and many limiting factors (darkness, oligotrophy, high mineral concentration) it was shown that microorganisms are able to thrive in the cave’s ecological niches. Highly specialized, perfectly adapted to this difficult ecosystem, cave microorganisms show vast biodiversity with countless novel species [27]. Bacteria, algae, and fungi were isolated from rock walls, cave soil, water, moonmilk, and vermiculation deposits [26,27]. Autotrophs generally draw energy by chemosynthesis, and organic and inorganic compounds from the rocks, cave sediments, and groundwater. Some microorganisms have also shown to utilize mixotrophy, while photosynthesis is available only for those microorganisms that live close to the light source, near the cave entrance. In any case, such microbial communities have been shown to take part in the formation of caves and sediments influencing several biogeochemical processes. They mainly act as promoters of precipitation or dissolution of minerals in the rock and cave water, which results in the emergence of new formations and speleothems [27,28,29].

To overcome growth limiting factors, microorganisms create complex, mutualistic networks. This strategy allows more organisms to survive and facilitates growth in extreme cave conditions [27]. Thick, multispecies biofilms composed of algae/cyanobacteriae, bacteria, or fungi are formed on cave walls and speleothems. It facilitates the flow of nutrients and allows multiple organisms to survive and thrive in this inhospitable environment [30]. However, some bacteria prefer competition from cooperation and produce secondary metabolites that inhibit the growth of other microorganisms in the vicinity, especially fungi [6,26]. Those are of particular interest for biotechnologists and medical microbiologists due to the potential use against drug resistant bacteria or fungi [6,31].

### 3.1. Photosynthesis and Lampenflora

In natural conditions, photosynthesis is only possible in most external parts of the cave [6]. Therefore, photosynthesizing algae and cyanobacteria are usually (but not only) present close to the cave entrance and the abundance of forms is higher there than in any other part of the cave [32]. Cyanobacteria and algae provide oxygen and organic compounds to be used by the heterotrophic part of the microbial community, frequently forming thick biofilms on cave walls [33,34]. Photosynthesizing microorganisms include members of cyanobacteria, chlorophyta, bacillariophyta, and rhodophyta, and the variations in relative abundance in these taxa is defendant both on the cave itself as well as the sampling point inside it [32,33]. For example, bacillariophyta and simple, trichal forms of algae are mostly present in biofilms formed nearest to the entrance [34,35], while coccoid and heterocystous cyanobacteria dominated biofilms show positive correlation with water availability inside the cave [33,34]. Research done by Czerwik-Marcinkowska et al. [36] in Ojców National Park, Poland showed, that autotrophic, green-pigmented organisms in samples consisted of 62% cyanobacteria, 21% green algae, and 17% diatoms, with *Gleocapsa* being a dominant cyanobacteria genus. The most frequently occurring diatoms were: *Diadesmis contenta* and *Luticola nivalis*, and they seemed to prefer naturally lit, permanently wet spaces. The diversity of photosynthesizing microorganisms in Ojców National Park caves seemed to be unaffected by the change of the seasons or other environmental factors, which corresponds with research done by other groups [17,36]. The four most frequent families of cyanobacteria in Ojców Park caves (Pseudanabaenaceae, Phormidiacae, Nostocaceae, Synechococcaceae) were also found to be dominant in cave samples from Ukraine [36]. Analysis of samples from caves in Italy done by Cennamo et al. [32] showed similar results as the Polish studies in terms of the abundance of the four main groups of autotrophs, with cyanobacteria being a dominant taxa, chlorophyta and bacillariophyta not exceeding 25% of algal population in each sample point, and rhodophyta never exceeding 10% of the autotroph population. However, dominant genera were different than these in caves of Central Europe [32,36]. Therefore, the climate, the dominant substrate of the rock, and other abiotic factors may be responsible for specific microclimatic differences, which will shape the microflora of the cave [32,37]. A large study done by Popović et al., 2020 [33] analyzed samples from 15 caves in Serbia. The extensive analysis focused not only on green pigmented autotrophs but also on algae and bacteria using other pigments to survive in deeper, darker parts of the cave. Green, photosynthesizing biofilms formed by chlorophyta were usually dominated by the genus *Desmococcus*. In bacillariophyta-dominated biofilms the genera were *Humidophyla*, *Gomphonema*, *Hantzschia*, *Luticola*, *Orthoseira* and *Pinnularia*, while the *Nostoc* taxa was the most popular representative of cyanobacteria. Furthermore, purple and red (dominated by *Gloeobacter* cf. *violaceus*), yellow (formed by *Gloeocapsa* spp.) and black (*Chroococcidiopsis* spp.) biofilms were observed in deepest, darkest areas of the studied caves [33].

**Figure 3:**
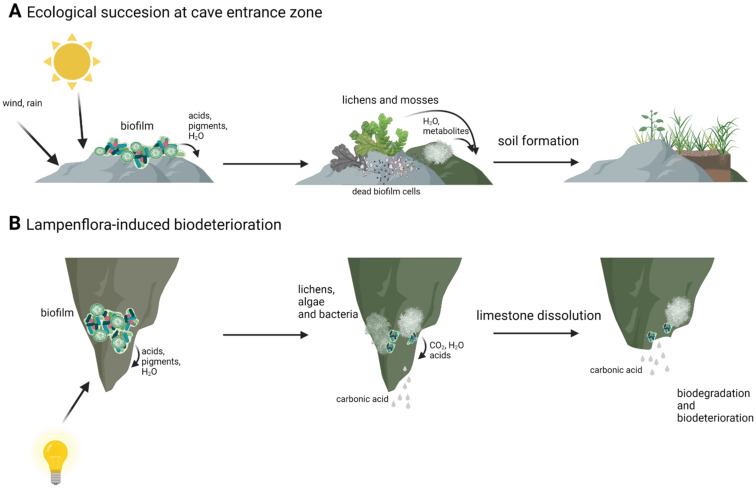
Differences between natural succession in cave entrance zone (**A**) and lampenflora-induced biodeterioration in cave dark zone (**B**). Created with BioRender.com (accessed on 4 January 2022).

Autotrophic algae are an important part of the cave ecosystem, as they reduce carbon dioxide and deliver oxygen and other substances to heterotrophic organisms as a result. The formation of multispecies biofilm also provides environmental stress protection, better binding of water, and different organic compounds, which can be very scarce in harsh cave environment and allows for the concentration and circulation of nutrients between organisms involved in biofilm [38,39,40,41]. This results in biofilm functioning as its own closed, little ecosystem. The presence of phototrophic microorganisms is also important for ecological succession in the most external cave zones. By secreting acids, pigments and other secondary metabolites microorganisms cause the deterioration and degradation of the rock. On subsoil made of degraded limestone and dead microorganisms, higher forms of phototrophic organisms such as lichens and mosses can develop. Finally, thin layers of soil are formed, which can be inhabited by higher plants (Figure 3) [38]. However, cave algae and cyanobacteria can be a source of interest for microbiologists and biotechnologists not only from an ecological point of view. Cyanobacteria can be a source of pigments, active secondary metabolites and toxins, and with extreme environments, such as caves still harboring poorly studied or unknown microorganisms there is a possibility for new substances to use in biotechnology [33,42]. Cyanobacteria isolated from various sources, including extreme environments, were shown to have potential to be used in medicine, as it was demonstrated that cyanobacterial extracts had antibacterial and anticancer properties [43,44]. Lamprinou et al., 2015 [45], showed that some of the cave cyanobacteria secreted antimicrobial compounds. However, in some cases it is the mixture of compounds that is responsible for antimicrobial activity and it is difficult or even impossible to pinpoint and extract potential medicine [43,44,46]. Furthermore, representatives of the genus *Chroococcidiopsis* are known to be able to withstand most extreme conditions, and therefore it is used in astrobiological studies [47,48].

However, these autotrophs and biofilms formed by them can also have a negative influence on cave ecology. Installation of artificial lighting in caves available to tourists resulted in the development of the so called “lampenflora” [15,49]. It is a community of phototrophs: algae, cyanobacteria, mosses, and ferns that form in the vicinity of the light installation [49]. Lampenflora is, contrary to other photoautotrophs existing in caves, completely independent from sunlight and other environmental factors. It is also hard to remove and expands quickly [37,49,50]. The development of artificial light associated with biofilms was first observed in show caves like Lascaux and Altamira, where unaesthetic drippings began to spread onto prehistoric paintings, reducing their visibility and causing its deterioration [15,21]. It was soon observed in other caves frequently visited by tourists, growing on illuminated paths through the caves and interesting rock formations [34,40,49]. Microbiological analysis of lampenflora biofilm samples revealed that in some cases it consisted of species already present in the cave: the spores and cysts of microorganisms, that were able to enter the vegetative state due to introduction of artificial light [33]. However, the composition of lampenflora differed from natural microorganisms present in the caves with *Chlorella* and *Trohiscia* being the dominant genera [18,35,39,49]. These differences in composition are thought to be caused by different growth conditions than those that exist for naturally occurring phototrophs in caves (i.e., the same wavelength and light intensity all year). Additionally, visitors are responsible for changes in humidity, CO_2_ concentration, and temperature changes. They may also act as carriers of foreign species of algae and bacteria, which would later develop along the touristic trail [15,17,33,41]. The presence of lampenflora is a problem not only of aesthetical nature, as it also disrupts the ecological balance of the cave, with some naturally occurring autotrophs having to fight for their survival [33,49,50]. Furthermore, microorganisms present in these biofilms have been found to be responsible for the deterioration of various types of substrate onto which it is attached [30,47]. Rock degradation and its transformation to soil is a naturally occurring process in places in caves that have access to natural light. However, biofilms associated with artificial light are responsible for biodeterioration in places when it would not occur naturally (Figure 3). Thus, the removal of lampenflora became an important issue in caves with frequent tourists visits [51,52]. Currently, the use of hydrogen peroxide, microwaves, and UV-C light are seen as the most promising and effective methods [35,52,53]. UV-C light has been reported to be able to kill microalgae with no new lampenflora development for long periods of time [35,54,55]. H_2_O_2_ and microwaves have also been reported to be effective against unwanted cave biofilms. Hydrogen peroxide has been shown to provide good results in terms of the eradication of algae and chlorophyll degradation [51]. However, prolonged use of hydrogen peroxide in carbonate formations can induce calcite dissolution [56]. Exposure to radiofrequency electromagnetic field also resulted in reduction of biofilm biomass ranging from 50% to 90% [53,57].

### 3.2. Biomineralization

During their life, microorganisms can produce H_2_O, CO_2_, as well as other compounds and metabolites that are removed from the cell. It has been observed that some of these compounds are responsible for processes known as biomineralization. It is the chemical alteration of the environment by microorganisms, resulting in the precipitation of minerals [58]. The process is not only limited to caves, as bacteria influencing crystal formation are also found in soil, and some pathogenic bacteria such as *Helicobacter pylori*, *Pseudomonas aeruginosa*, or *Proteus vulgaris* are also involved in crystal formation, being involved in the development of intracellular urinary stones [59,60]. The process of biomineralization can be biologically controlled—with microorganism synthesizing minerals at certain conditions at specified locations within or outside the cell [61]. It can be biologically influenced—a passive mineral precipitation caused by the presence of cell surface organic matter like extracellular polymeric substances (EPS) [58] or biologically induced—mineralization occurs as a result of metabolic activity of microorganism that leads to the precipitation of minerals in the surrounding environment [58,61]. Biominerals produced by microorganisms can be divided into three main mineral classes: carbonates, phosphates, and silicates [62]. However, other compounds such as sulfur and iron can also be utilized in biomineralization processes [63,64,65]. In terms of cave ecosystems, the minerals that are most commonly formed under the influence of microorganisms are carbonates [58,62]. Precipitation of carbonates is induced by bacteria via urea hydrolysis [66,67]. Urease activity influences chemical processes associated with mineral precipitation such as change in pH, dissolved inorganic carbon concentration, or the availability of nucleation sites [61]. Research done at Altamira Cave, Spain, showed the uptake of carbon dioxide by CO_2_ efflux measurements in “gray spots” (areas heavily colonized by bacteria, with visible biomineralization processes). This leads to change in pH and limestone dissolution. In periods of low humidity and/or low CO_2_ concentration generation, the release of Ca^2+^ into the solution results in mineral precipitation and the formation of CaCO_3_. Therefore, bacteria can modulate the levels of CO_2_ in their environment by the induction of crystal formation [20].

**Figure 4:**
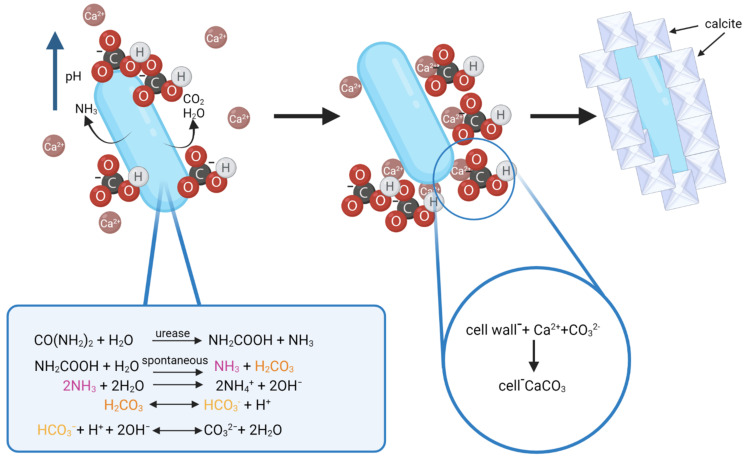
Schematic of biomineralization by urease producing bacteria. Source: Anbu et al., 2016 (modified) and Enyedi et al., 2020 (modified). Created with BioRender.com (accessed on 4 January 2022).

The process of calcite formation during biomineralization is thought to occur as follows: urease catalyzes the hydrolysis of urea into ammonia and carbamic acid, which is spontaneously hydrolyzed to another one mole of ammonia and carbonic acid. NH_3_ and H_2_CO_3_ are later equilibrated in water to form bicarbonate, ammonium, and hydroxide ions. Release of hydroxide ions leads to an increase of pH, which can shift the bicarbonate equilibrium, resulting in the formation of carbonate ions. This shift leads to the precipitation of metal ions. The generation of NH_4_^+^ further increases pH, HCO_3_^−^ ions get deprotonated and the CO_3_^2−^ content increases in the cell EPS matrix. The Ca^2+^ ions present in the environment (limestone) bind with the negatively charged bacterial cell wall, and this is where the precipitation of amorphous calcium carbonate (ACC) occurs as Ca^2+^ binds with CO_3_^2−^ [61,68]. As the cell dies, the disruption of the EPS and the cell wall opens up the ACC covering hydrophobic layers and gives rise to the formation of calcite [68]. Furthermore, CO_2_ and water released by bacteria as a byproduct of metabolic activity and present in the environment may influence the process by shifting the bicarbonate equilibrium in one way or the other during calcite formation [20]. The process of calcite formation is presented in Figure 4.

**Figure 5:**
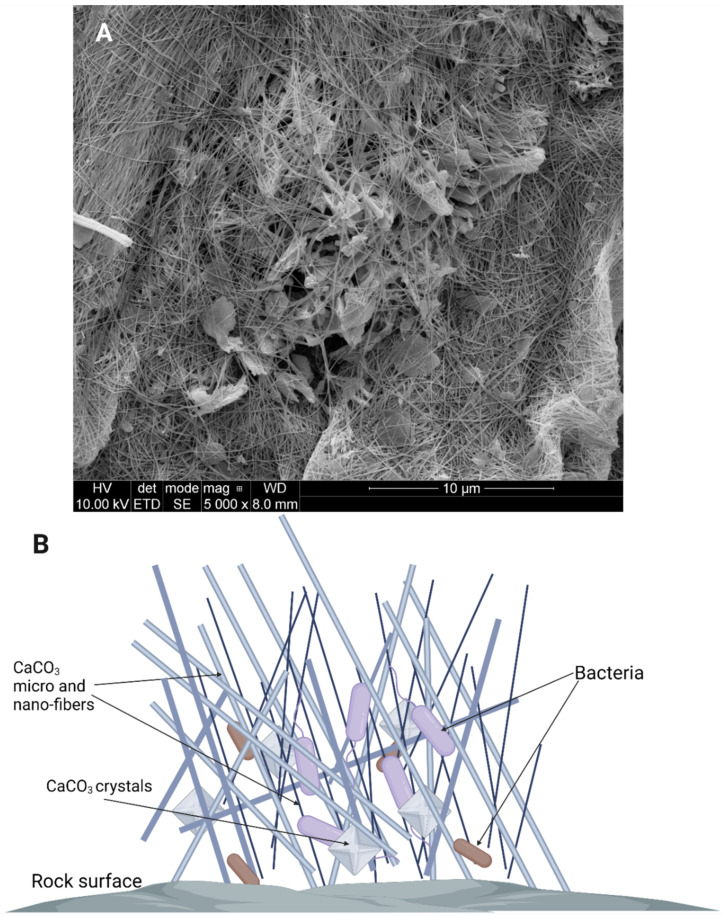
Moonmilk composition shown as images from SEM ((**A**) Source: Piotr Golec) and on a schematic (**B**). The SEM image was obtained from a moonmilk sample from Szczelina Chochołowska, Tatra Mountains, Poland, with the use of FEI Quanta 250 FEG Scanning Electron Microscope. The sample was obtained and prepared by Daria Lubomska. Created with BioRender.com (accessed on 4 January 2022).

The formation of crystals is one way microorganisms can influence the changes in their abiotic environment. Apart from calcite formation, bacteria can also influence rock dissolution and the formation of moonmilk and vermiculation. “Moonmilk”, a milky, white, mud-like exudate covering the surfaces in some caves, is primarily composed of fine CaCO_3_ nano- and micrometer sized needle-fiber crystals [69], with microorganisms being suspended, bound, or in other ways “tangled” with the crystals (Figure 5). The formation of moonmilk is attributed to actions of indigenous microorganisms. However, the mechanism of its creation is still unknown which leads to different hypotheses explaining moonmilk origins and the role microorganisms have in its formation. According to some, moonmilk is thought to have been created by the bacterium *Macromonas bipunctata*. However, no confirmation has been found to date [70]. Current studies suggest that Actinobacteria, especially *Streptomyces* spp., which are abundant in moonmilk samples influence its formation [71]. Analysis done by Maciejewska et al. [72], both in vitro and in vivo, suggest that in certain conditions *Streptomyces* spp. are able to induce cave bedrock dissolution. This phenomenon is related to the acidification of the microenvironment, most likely as a result of production of organic acids during microbial carbon metabolism. Organic acids decrease the pH of the bacterial surrounding leading to dissolution of limestone. Due to microbial urease activity in processes that are independent from carbon metabolism, the NH_4_^+^ is excreted from bacterial cells, leading to a local increase of pH. In these sites on the cell, where the pH in the environment is higher and CO_3_^2−^ accumulates in (EPS), are then used as nucleation sites for mineral precipitation and crystal formation [71]. Therefore, moonmilk is formed as a result of ongoing opposite processes as mineral dissolution occurs in one place, and then the same mineral is precipitated in others in the form of calcite [62,71,73]. Moonmilk composition and SEM images are presented in Figure 5. Vermiculations are deposits that form worm-like patterns on cave walls and they are represented by various morphologies, shapes, and colors and are generally composed of calcite, associated with quartz, and traces of clay minerals [74]. During studies carried out by various research groups it was discovered that, similarly to moonmilk, vermiculations are rich in microbial life forms [27,75]. It is also assumed that, similarly to moonmilk formation, vermiculation forms when bacteria dissolve minerals in one place and then precipitate other minerals in another [27,71]. Metagenomic studies suggested that members of *Proteobacteria*, Actinobacteria and cyanobacteria are abundant in this formations and that it is possible that the relations between these microorganisms and their life processes are what influence the type of vermiculation that is formed [27,76]. Proteobacteria are usually the dominant phylum, most likely due to a wide range of metabolic pathways that offer the capability to degrade a broad spectrum of organic substrates and is often associated with Fe-Mn deposits [63,77]. Actinobacteria may play a role in the dissolution and precipitation of carbonates, while sulfur reducing/oxidizing bacteria were found to be present in vermiculation with sulfur deposits [27]. Therefore, even though the processes involved in the formation of vermiculations are not fully described or known, it could be assumed that multiple different bacteria with the ability to dissolve and/or precipitate different minerals are involved in their formation. The type of process involved will also depend on the minerals available and on the conditions in the cave [27].

The ecological role of biomineralization, formation of vermiculations, and other mineral deposits is not clear. It is a way microorganisms shape their environment; however, in some cases it is seen as a side effect of bacterial metabolism rather than intentional impact. However, due to correlation of bacterial phyla seen especially in vermiculations as well as observed cooperation between cave microorganisms, it is not excluded that the processes of mineral dissolution carried out by one bacterial phylum is a source of compounds needed by another phylum for their metabolism [13,21,27].

The processes of mineral dissolution and precipitation by bacteria are sought to be utilized by man, especially in the construction industry. Biomineralizing bacteria could be used for soil stabilization, concrete remediation, and mineralization of cementitious materials. Use of microorganisms for these processes is seen as an alternative to conventional techniques such as applying cement or chemicals that are primarily used to improve soil quality, as these techniques have been linked with permanent soil and water contamination [61]. The production of cement has also been proven to have a negative impact on the environment, especially in terms of CO_2_ generation [78]. Utilization of natural, bacterial processes of mineral precipitation and soil binding should be deprived of the negative influence on the environment [73,78]. First small scale trials on raw materials and soil and sand samples have been carried out using soil-derived bacteria such as *Bacillus* sp. CT-5 or *Lysinibacillus sphaericus* WJ-8, and they have been successful [78,79,80]. It is also possible, that cave-derived biomineralizing bacteria may be used in these processes. However, the application on a larger scale is yet to be carried out and therefore the full potential of biocementation is not known [58,61]. Furthermore, the use of bacteria on a large scale, especially for soil and sand solidification, seem to have one disadvantage, which is time [61].

Another way biomineralizing bacteria could be utilized is for the binding of radionuclides and heavy metals. The disposal of radionuclide wastewater from nuclear plants is highly toxic to the environment, particularly to human health [81]. Different research groups utilized different physico-chemical processes such as chemical precipitation, ion exchange, membrane process, immobilization, or adsorption in order to remove radionuclides [61,81]. Fujita et al. [82] suggested the use of ureolytic bacteria for cleaning up the radionuclides safely from the environment. The method involves stimulation of ureolytic microorganisms to precipitate CaCO_3_, which in turn leads to promote co-precipitation of radionuclides (especially strontium) by substitution of Ca^2+^ ion and formation of radionuclide carbonate minerals. Strontium 90 exists in the environment as the Sr^2+^ ion, which has chemical similarity to Ca^2+^. This means that Sr^2+^ can replace calcium ions in living systems. Therefore, the introduction of biomineralizing bacteria and induction of calcite precipitation would lead to competition of Sr^2+^ and Ca^2+^ and should result in the formation of SrCO^3^ [83]. Trials suggest that up to 95% of strontium was captured and neutralized in the form of crystal with the use of ureolytic bacteria *Sporosarcina pasteurii* WJ-2 or *Halomonas* sp. SR4 [61,83]. The use of mineral precipitating microorganisms have also been suggested in the removal of heavy metals from the environment. However, most of the organisms used in first trials proved ineffective, as heavy metals affected their ability to form minerals. Therefore, several research groups have isolated metal tolerant microbes with ureolytic capability from various extreme environments such as caves or mine-shafts to improve the efficiency of the process [61,79,84]. The process of binding heavy metals is similar to the neutralization of radioactive strontium. Heavy metals such as lead, chromium, cadmium, or arsenic are used as substitutes for Ca^2+^ and bound in the form of carbonates by microorganisms in the process of mineralization [84,85].

The use of biomineralizing microorganisms in the construction industry as well as in the removal of toxic minerals from the environment is very promising. Caves seem to be promising sources of microorganisms with such characteristics [68,86]. Furthermore, other pathways of mineral precipitation by bacteria (such as sulfur or nitrogen) may be utilized in the future. Therefore, the isolation and characterisation of biomineralizing bacteria as well as studies on metabolic pathways that lead to precipitation should be carried out [58,61,73].

### 3.3. Antibacterial and Antifungal Secondary Metabolites

Among all properties displayed by cave microorganisms that may have potential applications, the use of secondary metabolites in medicine draws the most attention [6]. Due to antibiotic misuse and abuse we are currently facing a worldwide antibiotic crisis. More and more strains are reported to display resistance to many antibiotics at once [87]. Therefore, alternative methods of treatment against bacterial infections are being developed, as well as new antimicrobial compounds are being researched [88,89]. Extreme environments such as caves, glaciers, or deep seas seem to be promising sources of microorganisms with properties that could be implemented in medicine [31,90]. Secretion of secondary metabolites with antimicrobial properties by microorganisms living in extreme, oligotrophic environments such as caves is not surprising, as competition for resources is strong, especially in the deepest parts of the cave [6,91]. However, it is assumed that due to the complexity of metabolic pathways, the diversity of antimicrobial compounds secreted by a single strain and the reported synergy between the produced substances, their role may not only be limited to growth inhibition of competitive organisms, but may also play a role in interspecies communication [92,93] as cues triggering adaptations, such as motility or biofilm formation [91].

Studies on cave microbiomes revealed, that Actinobacteria, including *Streptomyces* genus, are an abundant phylum in caves. These bacteria are known for their complex metabolism production of various antibacterial and antifungal compounds as secondary metabolites [94,95]. It has been shown that *Streptomyces* are common in moonmilk, which has been popular in traditional medicine as it has been associated with healing and antibacterial properties since ancient times [96]. Therefore, moonmilk as well as cave water and other exudates have been areas of interest for microbiologists [9,26].

Over the last two decades, *Streptomyces* showing antibacterial and antifungal properties, have been isolated from moonmilk, soil, or water samples from caves all over the world, including Belgium [91], Poland [26], Serbia [97], Russia [98,99], China [100], India [101], Turkey [102], and Canada [103]. Actinobacteria other than *Streptomyces* have also been shown to produce metabolites with antibacterial potential against *Bacillus* spp., *Escherichia coli*, *Micrococcus luteus* and *Candida albicans* [104,105,106]. Furthermore, it was revealed that the production of antimicrobial compounds is not restricted to Actinobacteria. Research performed on samples from cave in Europe showed that members of *Proteobacteria*, *Bacteroidetes* and cyanobacteria also inhibit the growth of bacteria such as *Pseudomonas aeruginosa* or *Xanthomonas oryzae* and even VRE (Vancomycin Resistant *Enterococcus*) and MRSA (Methicillin Resistant *Staphylococcus aureus*) strains [45,107].

During research on cave microorganisms and their antimicrobial properties, few new compounds have been identified. Additionally, derivatives of already known antibiotics or pigments were described. Among substances isolated from cave microorganisms, there are, e.g., cervimycins A–D or polyketide glycosides isolated from *Streptomyces* sp. Strain HKI0179, which showed activity against MRSA and VRE. Cervimycins A and B are considered to be novel compounds, with cervimycins D and E being similar to compounds previously reported by Japanese research group [103]. Compounds named as hypogeamicins B-D isolated from *Nonomuraea* sp. Were described by Derewacz et al. [108] and showed antibacterial activity, particularly against *Bacillus subtilis*. Furthermore, xiakemycin A, a pyranonaphthoquinone, produced by *Streptomyces* sp. CC8-201 isolated from soil from karst cave located near Chongquing, China, was active against *Staphylococcus aureus*, *Staphylococcus epidermidis*, *Enterococcus faecalis*, and *Enterococcus faecium* with Minimal Inhibitory Concentrations ranging from 2 to 16 μg/mL [100]. *Streptomyces* sp. JS520 from Miroc Cave, Serbia, produced a red pigment, with antibacterial activity. Liquid chromatography and mass spectroscopy of the purified pigments revealed the major component to be undecylprodigiosin. The purified pigment was active against *M. luteus*, *B. subtilis*, *C. albicans* [97]. Another example of a bacterial pigment with strong antibacterial properties that can be found in cave ecosystems is violacein. This purple pigment was first isolated from soil-dwelling *Chromobacterium violaceum* and showed strong activity against Gram-positive bacteria [109,110]. Activity spectrum of violacein also included multi-drug resistant strains of nosocomial pathogens, and therefore this compound is seen as one of the future ways of treatment of MRSA or VRE strains [111]. Violacein and similar pigments have also been found to be produced by cave-dwelling members of *Janthinobacterium* genus, therefore caves may be a potential source of this antibacterial compound and its derivatives [112,113,114]. Other novel compounds identified in cave microorganisms are A12-C, a bacteriocin-like peptide from cave-derived *Bacillus licheniformis* that showed activity on *Bacillus megaterium*, *Corynebacterium glutamicum* and even *Mycobacterium*, as well as several fungi, such as *Microsporum canis*, *Mucor mucedo* and *Sporothrix schenckii* [115]. Lipids isolated from *Toxopsis calypsusstrain* ATHU-CY3314 and *Phormidium melanochrounstrain* ATHU-CY 3315—two cyanobacteria—also exhibited strong antimicrobial properties against Gram-positive bacteria [45].

However, in most cases it is the mixture of compounds or crude extracts that show antimicrobial activity, with a single compound being either unidentifiable or unknown [6]. Secondary metabolism of extremophilic bacteria can be very complex, with multiple compounds being released simultaneously and acting synergistically [116,117]. This results in a single isolated compound not showing any antimicrobial activity and needing the presence of additional compounds to boost its properties [118]. This was the case for samples from Bolshaya Oreshnaya Cave, Siberia, Russia. The mixture of four compounds: cyclodysidin D, chaxalactin B, stylissazole B, and gyrophoric acid, showed antibacterial (*B. subtilis*, *E. coli*, *P. putida*) and antifungal (*C. albicans*) properties [98]. Similarly, the case presented itself with isolates from Krubera-Voronja Cave, Georgia. Two isolates belonging to the family Baccillaceae showed high activity against Gram-positive microorganisms. In the case of both strains, the mixture of two compounds was responsible for antibacterial activity. Chemical analysis revealed that the main antibacterial compounds were pyrrolopyrazines and benzene derivatives [119]. Most recent studies report antibacterial activity of strains isolated from caves in Tatra Mountains [26], and in a 13,000-year old cave ice core in Siberia [93], but in those cases even though potential compounds have been identified through NMR or chromatography, more work is needed in order to isolate and fully characterized the compounds.

However, despite promising initial reports, the introduction of the cave bacteria-derived compounds to the medical market may be difficult and time-consuming. First, cave microorganisms live in specific environments and the recreation of optimal growth conditions in vitro is impossible. This leads to only a fraction of bacteria being isolated and cultivated in laboratories [9,91]. Furthermore, cultivation in vitro may result in changes in metabolism and thus: secondary metabolites secretion. Therefore, some of the compounds may be lost or their concentration may be too low for isolation and characterization procedures. This problem has already been reported by Jaroszewicz et al. [26] The *Streptomyces* isolated from a cave in Tatra Mountains, Poland, inhibited growth of multiple pathogens including MRSA and STEC (Shiga-toxin producing *E. coli*), but were unable to recreate the effect using culture extracts. Furthermore, since more than one compound can be responsible for antimicrobial effects, the isolation of these compounds may be difficult and time consuming. Therefore, production of the medicines based on cave bacteria metabolites may be a complicated process [6]. In order to fully benefit from the cave microorganisms’ antibacterial properties, more in depth studies need to be performed. The research should be complex and involve multidisciplinary analysis, combining microbiology, biotechnology, and chemistry. Detailed research on isolation procedures and growth conditions of novel strains should be done. Additionally, studies on metabolism regulation in antibiotic producing strains should also be performed in order to fully understand the mechanism and maximize compound production [6,112,117].

## 4. Summary

Extreme environments spark interest in scientists due to their uniqueness and inhospitable nature and the presence of organisms that are able to survive there, which display adaptation mechanisms that have not been seen anywhere else. Especially microorganisms seem to thrive in the difficult living conditions in these environments [6,120]. Research on the karst caves revealed great diversity of bacteria, algae, and fungi living on stone walls and speleothems, in mud puddles, or sediments. They have become the source of interest for various research groups including geologists, chemists, ecologists, or microbiologists [14,28,120]. The adaptations cave-dwelling organisms have applied to survive are complex, from multi-species biofilms that are able to function as closed, tiny ecosystems, through rock dissolution, to complex metabolic pathways, that utilize every element available. Some of the properties cave microorganisms display may have a potential use in various areas of human life [6,58]. Secondary metabolites produced by bacteria show strong antibacterial and antifungal properties. Discovery of novel antimicrobial compounds is important for medicine, as the current antibiotic crisis leaves us with fewer and fewer options for the treatment of bacterial diseases [26,102,111]. Compounds that could be isolated from cave microorganisms, especially Actinobacteria, could be turned into new medications [114,119]. The researchers even suggest that some of the secondary metabolites can even have anticancer and anti-inflammatory properties [26,31]. The search is not only limited to karst caves, as research done in lava and ice caves also reveals an abundance of microorganisms producing antibiotics or other antimicrobial compounds. However, more research is needed in order to identify, purify, and test these compounds and reveal their full potential [121,122]. Research on complex metabolic pathways of cave microorganisms is a potential source of new enzymes and compounds for biotechnology [95,116]. Furthermore, bacteria that are able to dissolve rock or induce mineral precipitation could be used in the construction industry and engineering. Bacteria could be used to stabilize soil in areas where the ground is considered too loose. Small scale trials with soil or mine-derived bacteria have even been made and the results are promising [58,73]. Taking this all into account, the exploration of extreme environments and studies on microorganisms inhabiting those environments could be beneficial on many levels and be a source of knowledge and solutions that could be implemented in many scientific, industrial, and medical areas. However, there are obstacles that need to be overcome in order to facilitate the research in these fields. Implementing metagenomics studies revealed an abundance of species living in caves, however, most of them still elude scientists as they are uncultivable in vitro [36,94]. Development of new media and isolation protocols should be made in order to increase the number and diversity of recovered strains. Furthermore, the methods of cultivation should be optimized in order to obtain higher concentration of bioactive metabolites [9,112]. Numerous active strains have been isolated from caves, but the bioactive compounds from only a few of them have been studied in detail. High-resolution methods of chemical analysis should be used more frequently and all unidentified compounds should be studied regardless of their activity, as seemingly inactive compounds have been discovered to be bioactive in the past [6,26,31]. Nevertheless, caves and other extreme environments are an interesting and promising source of various microorganisms and compounds with potential use. Therefore, the studies on this field, even though difficult due to characteristics of microorganisms and unique nature of the environments they inhabit, should prove rewarding.
